# Influence of metals and metalloids on the composition and fluorescence quenching of the extracellular polymeric substances produced by the polymorphic fungus *Aureobasidium pullulans*

**DOI:** 10.1007/s00253-020-10732-7

**Published:** 2020-06-23

**Authors:** Wenjuan Song, Yuyi Yang, Xinjin Liang, Feixue Liu, Geoffrey Michael Gadd

**Affiliations:** 1 State Key Laboratory of Desert and Oasis Ecology, Xinjiang Institute of Ecology and Geography, Chinese Academy of Sciences, Urumqi, 830011 China; 2 Geomicrobiology Group, School of Life Sciences, University of Dundee, Dundee, Scotland DD1 5EH UK; 3 Key Laboratory of Aquatic Botany and Watershed Ecology, Wuhan Botanical Garden, Chinese Academy of Sciences, Wuhan, 430074 China; 4 Present Address: Sustainable Environment Research Centre, Upper Glyntaff, University of South Wales, Pontypridd, Wales CF37 4BD UK; 5 State Key Laboratory of Heavy Oil Processing, Beijing Key Laboratory of Oil and Gas Pollution Control, College of Chemical Engineering and Environment, China University of Petroleum, Beijing, 102249 China

**Keywords:** Mercury, Selenium, *Aureobasidium pullulans*, Excitation–emission matrix (EEM) fluorescence spectra, Extracellular polymeric substances (EPS)

## Abstract

**Abstract:**

*Aureobasidium pullulans* is a ubiquitous and widely distributed fungus in the environment, and exhibits substantial tolerance against toxic metals. However, the interactions between metals and metalloids with the copious extracellular polymeric substances (EPS) produced by *A. pullulans* and possible relationships to tolerance are not well understood. In this study, it was found that mercury (Hg) and selenium (Se), as selenite, not only significantly inhibited growth of *A. pullulans* but also affected the composition of produced EPS. Lead (Pb) showed little influence on EPS yield or composition. The interactions of EPS from *A. pullulans* with the tested metals and metalloids depended on the specific element and their concentration. Fluorescence intensity measurements of the EPS showed that the presence of metal(loid)s stimulated the production of extracellular tryptophan-like and aromatic protein-like substances. Examination of fluorescence quenching and calculation of binding constants revealed that the fluorescence quenching process for Hg; arsenic (As), as arsenite; and Pb to EPS were mainly governed by static quenching which resulted in the formation of a stable non-fluorescent complexes between the EPS and metal(loid)s. Se showed no significant interaction with the EPS according to fluorescence quenching. These results provide further understanding of the interactions between metals and metalloids and EPS produced by fungi and their contribution to metal(loid) tolerance.

**Key points:**

*• Metal(loid)s enhanced production of tryptophan- and aromatic protein-like substances.*

*• Non-fluorescent complexes formed between the EPS and tested metal(loid)s.*

*• EPS complexation and binding of metal(loid)s was dependent on the tested element.*

*• Metal(loid)-induced changes in EPS composition contributed to metal(loid) tolerance.*

## Introduction

Toxic metal and metalloid pollution of soil and water can cause serious ecological and environmental problems. Microbial bioremediation has long been regarded as an environment-friendly way to treat such pollution. Microorganisms including bacteria, yeasts, filamentous fungi, and algae have been widely investigated for the removal and treatment of toxic metals (De Philippis et al. 2011; Fomina and Gadd 2014; Gadd 2010; Yin et al. 2019). The interactions between microorganisms and metals can involve several processes, e.g., adsorption, bioprecipitation, redox transformations, ion exchange, and complexation by excreted metabolites and extracellular polymeric substances (EPS) (Gadd 2000, 2004; Gupta and Diwan 2017; Sheng et al. 2013). The EPS secreted by microorganisms have been proven to be a rich matrix of polymers, including polysaccharides, proteins, glycoproteins, nucleic acids, phospholipids, and humic acids, which are thought to promote cell–cell recognition/communication and protect cells from adverse environmental conditions such as desiccation (Prabhakaran et al. 2016; Ravella et al. 2010; Zhang et al. 2015). Furthermore, EPS is believed to play an important role in the tolerance of microorganisms exposed to toxic metals acting as a protective barrier and also a matrix for geochemical transformations and bioprecipitation (Fomina et al. 2005; Hou et al. 2013; Naik et al. 2012). This has been observed in a variety of microorganisms, such as sulfate-reducing bacteria (Yue et al. 2015), iron-oxidizing bacteria (Liu et al. 2017), and the fungi *Phanerochaete chrysosporium* (Cao et al. 2018) and *Beauveria caledonica* (Fomina et al. 2005).

*Aureobasidium pullulans* de Bary (Arnaud) is a polymorphic fungus and can exist as yeast-like cells, hyphae, intermediate cellular and filamentous forms, and melanized chlamydospores (Gadd and Griffiths 1980; Gadd 1980, 1984). It is ubiquitous and widely distributed in the environment, and can be isolated from soil and water, and is an early-colonizing saprophyte on decaying leaves and various surfaces such as concrete, wood, leaves, and tree bark (Bhadra et al. 2008; Prasongsuk et al. 2018). *A. pullulans* shows substantial tolerance against toxic metals, e.g., lead, copper, nickel, and cadmium (Deshpande et al. 1992; Gadd and Mowll 1985; Mowll and Gadd 1984; Nakkeeran et al. 2018). The ability of *A. pullulans* to form melanized chlamydospores and hyphal structures can play a significant role in protection, acting as an efficient barrier for metals, preventing intracellular uptake, and able to accumulate large amounts of metals by biosorption (Gadd 1984; Gadd et al. 1984; Mowll and Gadd 1984). In the environment, *A. pullulans* can become the dominant organism on phylloplanes contaminated by aerially deposited lead and other metals (Bewley and Campbell 1980; Mowll and Gadd 1985). *A. pullulans* strains showed significant Pb tolerance when grown on solid media containing 7.5 mM Pb^2+^ (Rhee et al. 2014) and able to remove Pb, and other metals including Cd, from solution by biosorption and other accumulation mechanisms (Mowll and Gadd 1984; Radulovic et al. 2008). *A. pullulans* extensively synthesizes a mixture of extracellular pullulan-based polysaccharides and protein, which may enhance environmental survival and metal tolerance, being thought to play an important role in metal detoxification (Cheng et al. 2011; Suh et al. 1999). It was observed that the presence of EPS could influence adsorption of Pb^2+^ on the cell surfaces of *A. pullulans* and inhibit direct interactions with cellular components (Suh et al. 1999). However, the extracellular polysaccharide (pullulan) produced by *A. pullulans* CH-1 apparently does not bind Pb or other metals, including Cu, Fe, Zn, Mn, Cd, Ni, and Cr (Radulovic et al. 2008).

Although the extracellular polysaccharide of *A. pullulans* and its significance for tolerance to toxic metals has been examined (Čertík et al. 2005; Mowll and Gadd 1984), information is limited and often contradictory, while the role of protein in fungal EPS has not been fully ascertained. Furthermore, there have been no reports focusing on the behaviour of *A. pullulans* when exposed to mercury or metalloids, e.g., arsenic and selenium. The aim of the present work was to study the effects of Pb^2+^, Hg^2+^, As^3+^, and Se^4+^ on the growth of *A. pullulans* and EPS production and composition, and to investigate complexation of EPS with these metals and metalloids using fluorescence spectroscopy. The results obtained shed further light on the potential interactions between metal(loid) elements and *A. pullulans*, and the significance of EPS in such interactions and metal tolerance.

## Material and methods

### Microorganism and growth medium

*Aureobasidium pullulans* de Bary (Arnaud) (IMI 45533) was sub-cultured from the Geomicrobiology Group collection, School of Life Sciences, University of Dundee. The strain was maintained on EPS production medium (EPM) consisting of the following (g L^−1^): sucrose 50; peptone 0.6; yeast extract 0.4; K_2_HPO_4_ 5.0; MgSO_4_·7H_2_O 0.4; NaCl 1, which were mixed and dissolved in Milli-Q water. The pH of the medium was adjusted to pH 6.0 using 1 M HCl or NaOH and then autoclaved at 115 °C for 15 min before use.

### Solution preparation

Two metal elements (Hg and Pb) and two metalloid elements (As and Se) were chosen. Mercury and lead stock solutions were prepared using mercuric chloride (HgCl_2_) and lead nitrate (Pb(NO_3_)_2_), respectively. Sodium arsenite (NaAsO_2_) and sodium selenite (Na_2_SeO_3_) were used to prepare the arsenic and selenium stock solutions. All solutions were sterilized by filtering through 0.45-μm cellulose nitrate membrane filters (Minisart syringe filters, Sartorius, Göttingen, Germany) and stored at 4 °C prior to use.

### Effect of metals and metalloids on growth of *A. pullulans*

All experiments were carried out using EPM medium. As exogenous stress factors, 0.01 and 0.1 mM Hg, 0.1 and 1 mM Pb, 1 and 10 mM As, and 1 and 10 mM Se were incorporated into the media. These stress factors were added at the beginning of the growth experiments when flasks were inoculated from an exponentially growing liquid starter culture of *A. pullulans*. The experimental flasks were inoculated to an initial OD_600_ of 0.3 from this starter culture and were incubated at 25 °C for 96 h in a shaking incubator (120 rpm). Tolerance indices (TI) were used to compare optical density values in EPM medium with or without the stress factors. A TI value lower than 1 indicated growth inhibition; a TI value larger than 1 suggested growth stimulation (Sayer et al. 1995).

### Extraction and purification of EPS

EPS was extracted by high-speed freezing centrifugation (Chu et al. 2015; Zhao et al. 2015). Briefly, cultures were centrifuged at 4 °C (2012*g*, 20 min) to obtain cell pellets and an EPS-containing supernatant. After that, the supernatant was purified by dialysis using a 3500 Da membrane for 24 h at 4 °C, and then the EPS precipitated with two volumes of ethanol (95%). The ethanol was added slowly into the supernatant with constant stirring, stored at 4 °C overnight, and the precipitate collected by centrifugation at 4 °C (2012*g*, 20 min). Cell pellets (after washing with MilliQ water) and EPS were collected and dried at 50 °C to constant weight. EPS yield was normalized as mg EPS per g cell dry weight.

### Analysis of biochemical components of EPS

Polysaccharide content was measured using the phenol-sulfuric acid method, using glucose as the standard (Nielsen 2010). Protein content was measured using the Bradford method, with bovine serum albumin as standard (Kruger 2009).

### Excitation–emission matrix fluorescence spectroscopy: fluorescence quenching titrations

All excitation–emission matrix (EEM) spectra were obtained using a fluorescence spectrophotometer (F-7000, Hitachi, Japan) equipped with a 1.0-cm quartz cell and a thermostatic bath (Song et al. 2016b). EEM spectra were collected by scanning emission wavelengths from 200 to 500 nm in 2-nm increments by varying the excitation wavelengths from 200 to 450 nm in 5-nm increments. The width of the excitation/emission slit was set to 5 nm and the scanning speed was set to 1200 nm/min. All EPS sample were diluted 10 times and titrated with incremental microliter additions of 10 mM metal(loid) (Hg, Pb, As, and Se) solutions at 25 °C. After each addition, the EPS solution was fully mixed using a magnetic stirrer for 15 min and the fluorescence spectra recorded. The fluorometer’s response to a Milli-Q water blank solution was subtracted from the fluorescence spectra recorded for EPS samples under the same conditions. All titration experiments were carried out in triplicate and the mean values calculated. EEM data were processed using Sigmaplot 10.0 software (Systat, USA).

## Results

### Effect of metals and metalloids on growth of *A. pullulans*

**Fig. 1:**
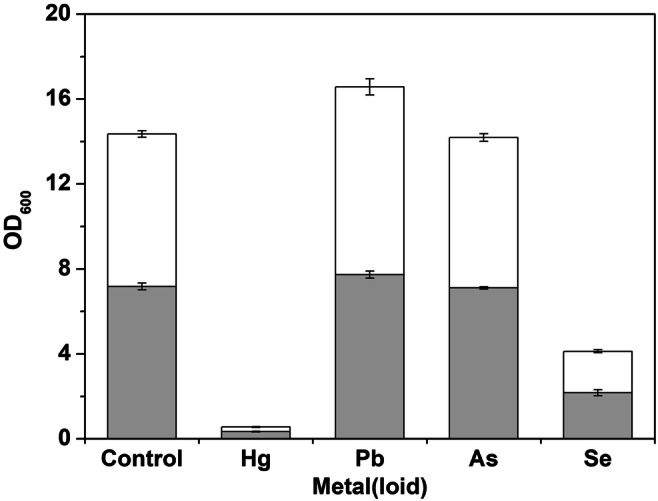
Optical density (at 600 nm) of *A. pullulans* cell suspensions after incubation for 96 h in the absence or presence of metal(loid)s at high () or low () concentrations (high concentrations of Hg, Pb, As, and Se were 0.1, 1, 10, and 10 mM, respectively: low concentrations of Hg, Pb, As, and Se were 0.01, 0.1, 1, and 1 mM, respectively). Bars are the standard deviation of average values

The optical density of cell suspensions at 600 nm (OD_600_) was used to assess growth in the presence of different metals and metalloids. Growth of *A. pullulans* at different concentrations of metals and metalloids after 96-h incubation is shown in Fig. 1. Cell growth was significantly inhibited by Hg and Se. As caused little inhibition of growth of cells, while low concentrations of Pb (0.1 mM) had no effect on growth. A higher Pb concentration (1 mM) slightly increased the optical density which is thought to be a result of lead precipitation with growth medium components. Calculation of TI values confirmed these results. Mercury exhibited the highest toxicity to *A. pullulans* at low (TI = 0.05) and high concentrations (TI = 0.03). Selenium also significantly inhibited the growth of *A. pullulans* at low (TI = 0.30) and high concentrations (TI = 0.27). Lead slightly enhanced the growth of *A. pullulans* both at low (TI = 1.07) and high concentrations (TI = 1.23). Arsenic slightly inhibited growth of *A. pullulans* at low (TI = 0.99) and high concentrations (TI = 0.99).

### Effect of metals and metalloids on the yield and composition of EPS from *A. pullulans*

**Fig. 2:**
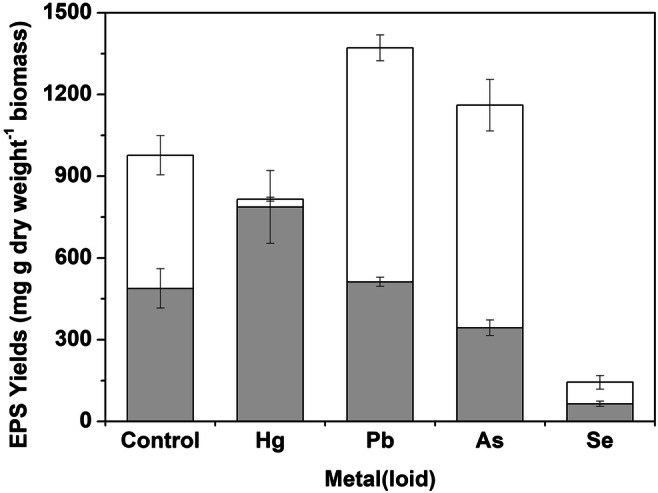
EPS yields from *A. pullulans* incubated for 96 h in the absence or presence of metal(loid)s at high () or low () concentrations (high concentrations of Hg, Pb, As, and Se were 0.1, 1, 10, and 10 mM, respectively: low concentrations of Hg, Pb, As, and Se were 0.01, 0.1, 1, and 1 mM, respectively). Bars are the standard deviation of average values

Figure 2 shows the EPS yields at different concentrations of metals and metalloid elements. *A. pullulans* in control EPM could produce EPS at 488.4 mg g^−1^ dry weight biomass. At the low concentration of the tested elements, mercury produced the highest amount of EPS with a value of 787.4 mg g^−1^ dry weight biomass, followed by Pb, As, and Se. Under mercury stress, the apparent high production of EPS but negligible cell growth might be attributable to the release of lipids and nucleic acids via cell lysis due to the toxicity of mercury particularly in the later stages of cell culture. At the high concentration of the tested elements, the Pb treatment showed the highest yield of EPS (858.9 mg g^−1^ dry weight biomass), followed by As (816.5 mg g^−1^ dry weight biomass).

**Fig. 3:**
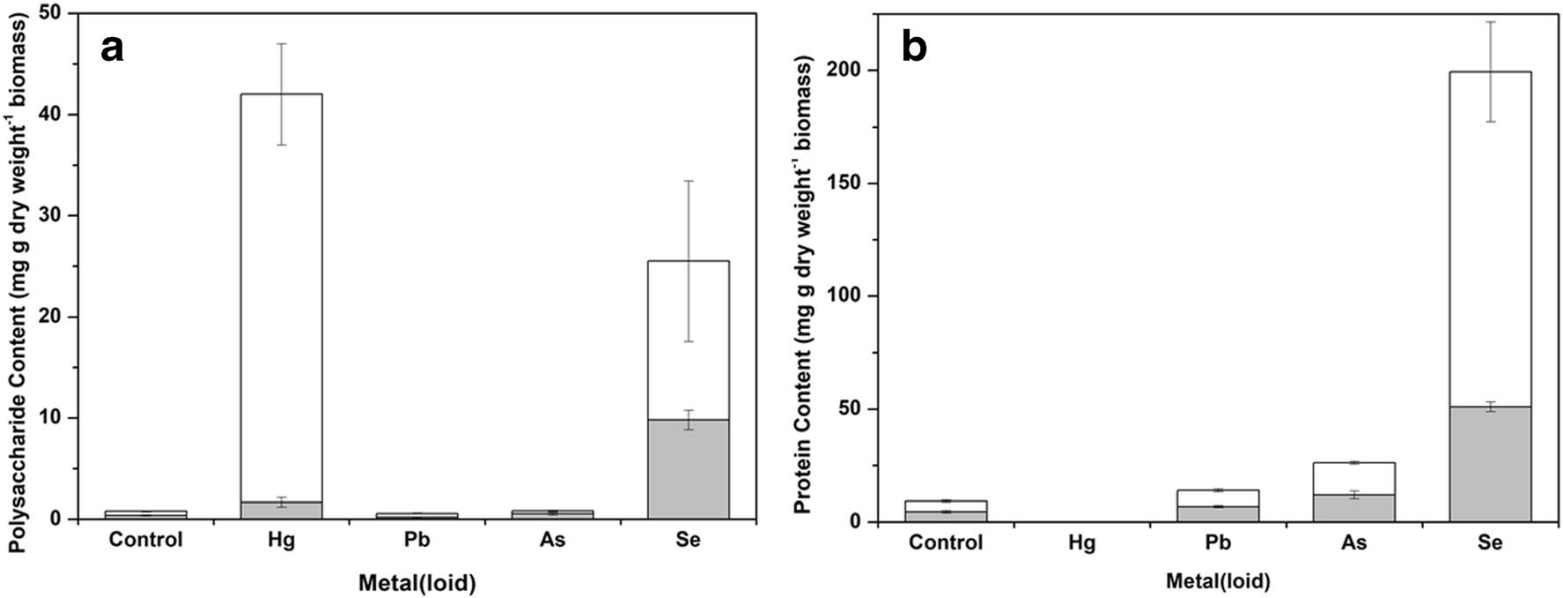
Polysaccharide (**a**) and protein (**b**) content of the EPS from *A. pullulans* incubated for 96 h in the absence or presence of metal(loid)s at high () or low () concentrations (high concentrations of Hg, Pb, As, and Se were 0.1, 1, 10, and 10 mM, respectively: low concentrations of Hg, Pb, As, and Se were 0.01, 0.1, 1, and 1 mM, respectively). Bars are the standard deviation of average values

The chemical composition of EPS is very heterogeneous and mainly composed of carbohydrates, proteins, lipids, and nucleic acids. Figure 3 shows the content of polysaccharide and protein in EPS from *A. pullulans* incubated with or without metals and metalloids. The content of protein (4.62 ± 0.50 mg g dry weight^−1^ biomass) was much greater than that of polysaccharide (0.26 ± 0.09 mg g dry weight^−1^ biomass) in the *A. pullulans* EPS incubated without metal(loids). It was also found that the polysaccharide content in the presence of Pb and As was similar to that of the control (Fig. 3a). The highest content of polysaccharide in the EPS occurred with mercury stress at the higher concentration, followed by the selenium treatment at both low and high concentrations. Regarding the protein content, the presence of selenium resulted in a higher protein content compared with the other tested elements (Fig. 3b). The Pb and As treatments at both low and high concentrations showed a slight increase in protein content compared with the control. However, negligible protein was found in the EPS with mercury. These results indicated that Pb and As did not significantly change the composition of the EPS. However, Hg and Se significantly changed the composition of the EPS compared with the control, which may reflect the toxicity of these elements towards *A. pullulans*.

### Fluorescence characteristics of EPS from *A. pullulans*

Two fluorescence peaks were observed in the EEM spectra of the EPS. Peak A (Ex/Em, 275–280/330–340) and peak B (Ex/Em, 225/330–340) are attributable to tryptophan-like and aromatic protein-like fluorophores, respectively. The excitation /emission wavelength of the fluorescence peaks showed a slight blue/red shift, indicating that the presence of metals in the media had no influence on the type of fluorescent components of the excreted EPS (Table 1). The fluorescence intensity of EPS from *A. pullulans* grown in media amended with metals clearly increased, indicating that the presence of metals stimulated the production of extracellular tryptophan-like and aromatic protein-like substances. The higher metal concentrations stimulated a greater production of fluorescent substances.

**Table 1:** Fluorescence position (Ex/Em, nm) and intensity (arbitrary units) of EPS from *A. pullulans* in the absence and presence of metal(loids). Intensities are average values from several determinations

Stress factor		Fluorescence peak and intensity			
		Peak A		Peak B	
Metal(loid)	Concentration (mM)	Ex/Em	Intensity	Ex/Em	Intensity
Control		280/335	586.4	225/330	879.8
Hg^2+^	0.01	275/335	1387	225/335	1966
	0.1	280/335	1485	225/335	2139
Pb^2+^	0.1	280/330	873.6	225/325	1231
	1	280/340	1025	225/330	1212
As^3+^	1	280/335	833	225/330	1283
	10	280/340	1998	225/340	2232
Se^4+^	1	280/335	1141	225/335	1906
	10	280/330	1585	225/330	2105

### Fluorescence quenching

**Fig. 4:**
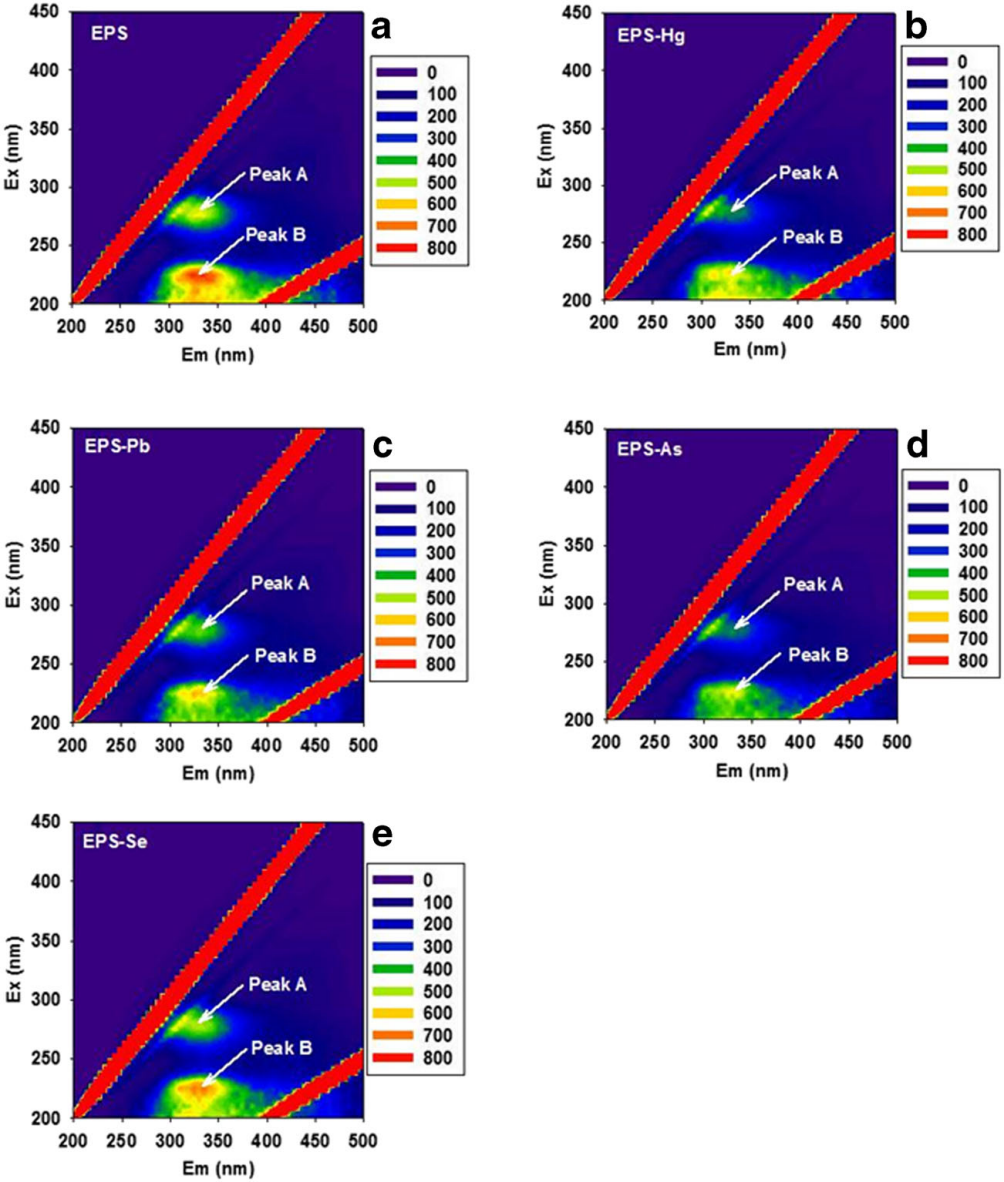
Typical three-dimensional fluorescence EEM spectra (Ex and Em, nm) of EPS at 25 °C in the absence and presence of different metal(loid)s. **a** EPS; **b** EPS + Hg; **c** EPS + Pb; **d** EPS + As; **e** EPS + Se). The experimental concentration ranges of detected metal(loid)s for EEM were 0–0.15 mM (Hg); 0–0.11 mM (Pb); 0–0.15 mM (As); and 0–0.1 mM (Se). Typical spectra are shown from several determinations

Three-dimensional fluorescence spectra of EPS and the EPS-metal system are shown in Fig. 4. The fluorescence intensity of peaks A and B decreased significantly after addition of Hg, Pb, and As indicating that these three metal(loid)s have a strong interaction with the EPS and thus quench its fluorescence. The fluorescence intensity of both peaks showed a slight decrease after addition of Se, indicating that Se has little influence on EPS fluorescence.

The fluorescence quenching process can be interpreted as dynamic quenching, which is due to collisions between the fluorophore and quencher, or static quenching due to complexation between the fluorophore and the quencher. The Stern–Volmer Eq. (1) was used to judge whether the quenching processes observed here were dynamic or static:

1 $$ {F}_0/F=1+{k}_{\mathrm{q}}{\tau}_0\ \left[\mathrm{Q}\right]=1+{K}_{\mathrm{sv}}\ \left[\mathrm{Q}\right] $$

where *F*_0_ and *F* represent the fluorescence intensity in the absence and presence of the quencher, respectively. *k*_q_ is the quenching rate constant; *K*_sv_ is the quenching constant; *τ*_0_ is the average lifetime of the fluorescence in the absence of the quencher, which is taken as 10^−8^ s; and [Q] is the metal concentration.

Figure 4 shows that within the tested Hg, Pb, and As concentration ranges, the change of fluorescence intensity agreed well with the Stern–Volmer equation (Table 2). Furthermore, the quenching rate constant (*K*_q_) values for Hg, Pb, and As to EPS were one order of magnitude larger than the maximum diffusion collision quenching rate constant (2.0 × 10^10^/M/s) for a variety of quenchers with biopolymers, indicating that the fluorescence quenching process was mainly governed by static quenching by the formation of complexes.

**Table 2:** Stern–Volmer fluorescence quenching constants, *K*_sv_, and quenching rate constant, *K*_q_, of EPS from *A. pullulans* in the presence of different metals (Hg concentration range 0–0.15 mM; Pb concentration range 0–0.11 mM; As concentration range 0–0.15 mM; Se concentration range 0–0.1 mM; *R*^2^, determination coefficients)

Quencher		Quenching constants *K*_sv_ (× 10^3^/M)	Quenching rate constants *K*_q_ (× 10^11^/M/s)	*R*^2^
Hg	Peak A	2.61	2.61	0.8813
	Peak B	3.14	3.14	0.8650
Pb	Peak A	2.30	2.30	0.8329
	Peak B	2.18	2.18	0.7548
As	Peak A	2.69	2.69	0.9368
	Peak B	3.12	3.12	0.9586
Se	Peak A	0.67	0.67	0.3196
	Peak B	0.87	0.87	0.5108

### Binding constants and binding sites

For ligand molecules that bind independently to a set of equivalent sites on a macromolecule, the equilibrium between free and bound molecules is given by the Hill Equation (2):

2 $$ \log \left[\left({F}_0-F\right)/F\right]=\log\ {K}_{\mathrm{b}}+n\log \left[\mathrm{Q}\right] $$

where *F*_0_ and *F* are the fluorescence intensities in the absence and presence of the quencher, respectively. *K*_b_ is the binding constant, *n* is the number of binding sites, and [Q] is the metal concentration. The binding constants (*K*_b_) reflect the interactive intensity between the EPS and the metal.

The binding constants (log *K*_b_) and the number of binding sites (*n*) of the EPS-metal system are listed in Table 3. The maximum value of log *K*_b_ was obtained from the EPS-Hg(II) system at peak A, indicating that the tryptophan-like substances had a stronger ability to bind Hg(II). The log *K*_b_ and *n* of tryptophan-like substances (peak A) for the metals and metalloids both showed the same order of Hg > Pb > As. Mercury had the higher log*K*_b_ and *n* for aromatic protein-like substances (peak B) with metals, followed by As and Pb. For the same metal or metalloid, the log *K*_b_ and *n* of tryptophan-like substances (peak A) were higher than those for aromatic protein-like substances (peak B), indicating that the tryptophan-like substances had a stronger binding ability for Hg, As, and Pb than aromatic protein-like substances. However, EPS showed no binding ability for Se. These results implied that Hg, Pb, and As could interact with fluorescent components, resulting in the formation of a stable non-fluorescent complex between the EPS and the metal/metalloid species.

**Table 3:** Binding constants (log *K*_b_) and number of binding sites (*n*) for *A. pullulans* EPS complexation with different metals (Hg concentration range 0–0.15 mM; Pb concentration range 0–0.11 mM; As concentration range 0–0.15 mM; Se concentration range 0–0.1 mM; *R*^2^, determination coefficients)

Metal(loid)		Binding constant (log *K*_b_)	Binding sites (*n*)	*R*^2^
Hg(II)	Peak A	6.76	1.89	0.9065
	Peak B	6.08	1.69	0.8887
Pb(II)	Peak A	4.00	1.17	0.7984
	Peak B	1.08	0.42	0.7236
As(III)	Peak A	2.61	0.79	0.9369
	Peak B	2.18	0.66	0.9557

## Discussion

Toxic metals can exert a variety of effects on the morphology and physiology of fungi, including *A. pullulans* (Gadd and Griffiths 1980; Gadd and Mowll 1985; Gadd et al. 1986; Newby and Gadd 1987; Ramsay et al. 1999; Fomina et al. 2000). In this study, mercury and selenium had a negative effect on the growth of *A. pullulans*, but lead and arsenic had no significant influence on the growth of *A. pullulans* at the concentrations tested*.* At higher concentrations of lead, it even appeared to stimulate growth of this fungus as measured by OD_600_, although this was probably a result of lead precipitation as sulfate/chloride which are present as medium components (Gadd and Griffiths 1978; Gadd 1992). The other results indicated that the effects of the test metal(loid)s on the growth of *A. pullulans* depended on the concentration and the element concerned. Growth inhibition by selenium in liquid EPM media was more pronounced than results obtained from growth on solid agar media (Liang et al. 2019), probably reflective of binding interactions in the solid medium that can result in alleviation of toxicity (Gadd and Griffiths 1978; Gadd 1992). *A. pullulans* can exhibit a high biosorption capacity for lead (Suh et al. 2001). This is unsurprising since Pb has a very strong binding affinity for biomass components and, as mentioned, is also likely to precipitate out of solution as e.g. phosphate, chloride, or sulfate, which will also associate with biomass. Because of such effects, Pb bioavailability is frequently low and this can underlie such tolerance to apparently high Pb concentrations. Furthermore, the melanized walls of chlamydospores and other cell forms of *A. pullulans* show high metal binding and act as an impermeable barrier to toxic metals (Gadd 1984; Gadd et al. 1984; Mowll and Gadd 1984). Such properties and Pb chemistry explain why *A. pullulans* is often the dominant organism on phylloplanes contaminated with industrial or vehicular Pb emissions (Bewley and Campbell 1980; Mowll and Gadd 1985). Because of the ease of high biomass generation and metal tolerance, *A. pullulans* has been suggested to be a useful organism for certain metal bioremediation applications (Nakkeeran et al. 2018).

Exopolysaccharides and proteins are often reported to have important roles in complexation of metals in solution (Comte et al. 2008; Guibaud et al. 2009; Huang et al. 2011). In this study, the extracellular protein content was higher than that of the exopolysaccharides in the EPS produced in the presence of Pb, As, and Se. This result is different to some other studies on the composition of EPS from other microorganisms, such as *P. chrysosporium*, which showed a higher content of polysaccharide than protein in the EPS (Li et al. 2017). However, another study found that the EPS from an algal biofilm had a higher content of protein than polysaccharide (Ma et al. 2018). It has also been observed that the ratio of protein to polysaccharide in the EPS from *Acidithiobacillus ferrooxidans* was significantly influenced by the pH of media (Song et al. 2016a). The yeast *Rhodotorula mucilaginosa* was also found to secrete more polysaccharides when exposed to Pb^2+^ at high concentrations (1000–2500 mg/L) (Li et al. 2019) while a higher protein content in EPS was detected in sludge exposed to lead and copper (Jang et al. 2001; Wang et al. 2015). Arsenic stress was also found to increase the protein and carbohydrate content in EPS from bacteria isolated from industrial wastewater (Saba et al. 2019). Changes in the composition of EPS under Hg and Se stress may therefore contribute to the tolerance of *A. pullulans* to toxic metals by affecting metal bioavailability: metals have high binding affinities to amino acids, peptides, and proteins (Mejáre and Bülow 2001). Furthermore, many toxic metals can cause lysis of cell membranes and leakage of intracellular content (Gadd et al. 1986) which would also influence the yield and composition of EPS as measured in this study. This is particularly likely to have occurred with Hg.

Fluorescence spectroscopy is a powerful technique for identification of the components of EPS and their interactions with metals. In this study, Hg, Pb, and As showed a strong interaction with tryptophan-like and aromatic protein-like fluorophores, while Se had no obvious interaction with these components. Some other studies have described the interaction of EPS with mercury, lead, and arsenic using fluorescence spectroscopy and have obtained similar results. The fluorescence of EPS produced by activated sludge (Song et al. 2012), pure bacterial cultures (Baldi et al. 2017), and an algal biofilm (Ma et al. 2018) was all quenched by Pb. The fluorescence of EPS from an algal biofilm and pure bacterial cultures was also significantly quenched by Hg (Song et al. 2012; Zhang et al. 2010) and As (Lin et al. 2018). It is possible that siderophore production might contribute to fluorescence, but this was not investigated in this study: *A. pullulans* is capable of siderophore excretion (Wang et al. 2009). FTIR analysis of bacterial EPS also revealed the involvement of phosphate, carboxyl and hydroxyl, or amine functional groups from polysaccharides and proteins in the interactions with lead (Song et al. 2012) and arsenic (Saba et al. 2019). Some previous studies also demonstrated the involvement of protein in the EPS of a cyanobacterial biofilm in complexation of mercury and arsenic (Lin et al. 2018; Ma et al. 2018; Zhang et al. 2015).

The quenching constants of EPS for metals and metalloids were in the range 0.67–3.14 in this study. These values were different from those recorded for an algal biofilm, activated sludge, and certain bacterial strains (Lin et al. 2018; Ma et al. 2018; Song et al. 2016b; Zhang et al. 2010). The quenching rate constant value for Pb and EPS from the algal biofilm was between 3.61 and 8.54 (Ma et al. 2018), which is greater than that found in this study. It has been found that the quenching process of tryptophan-like substances by As belonged to dynamic quenching, but aromatic protein-like substances showed complexation with As (Lin et al. 2018). Furthermore, the *K*_q_ values for Se interactions with EPS in this study were one order of magnitude smaller than 2.0 × 10^10^/M/s, indicating that the fluorescence quenching process was dominated by dynamic quenching due to intermolecular collisions. However, the quenching process between chemically synthesized SeNPs and EPS from the selenite-reducing bacterium *Citrobacter freundii* Y9 combined both static and dynamic quenching (Wang et al. 2018). These results imply that both the microbial species and elemental form might affect the interactions with EPS. The binding constants (log *K*_b_) for metals and metalloids to the EPS from *A. pullulans* were in the range 1.08–6.76 that are consistent with previous studies. It was found that the binding constant value, log *K*_b_, was 3.45–4.52 for Pb to EPS from an algal biofilm (Ma et al. 2018), and 6.15–7.77 for that from activated sludge (Song et al. 2012). The binding constant value, log *K*_b_, was 2.22–3.82 for As to EPS from an algal biofilm (Lin et al. 2018). The maximal value of the binding constant was 3.14 for Hg to EPS from *A. ferrooxidans* at pH 8, and 7.06–7.09 for cyanobacterial EPS from a *Chroococcus* sp. (Song et al. 2016a, b). From the above, it can be concluded that the complexation and binding ability of microbial EPS to metal/metalloid elements is strongly dependent on the microbial species and the particular element involved. Overall, it can also be concluded that changes in the composition of EPS induced by potentially toxic metal(loid)s, which affect metal bioavailability, can contribute to metal tolerance and survival.
